# Utilization of cold-formed steel-framed emergency shelter for typhoon reliefs in the Philippines

**DOI:** 10.1016/j.heliyon.2024.e40244

**Published:** 2024-11-08

**Authors:** Daniel Nichol Valerio, Cheryl Lyne Roxas, Kenneth Jae Elevado, Jeremy Brian Branzuela, Desiree Dale Chua, Gabriel Lambatin, Gian Carlo Tiu

**Affiliations:** Department of Civil Engineering, De La Salle University, 2401 Taft Avenue, Manila, Philippines

**Keywords:** Cold-formed steel, Post-disaster response, Emergency shelters, Cost sustainability assessment

## Abstract

The destructive aftermath of storms and typhoons in the Philippines more prominently affects isolated remote locations that lack methodical and strategic procedures to address the situation. One form of assistance provided during disasters is the construction of shelters, which can be categorized as emergency, temporary, or transitional, serving as protection during recovery periods. Cold-formed steel (CFS) is widely used, and its versatility in fabrication and configurability make it an ideal candidate for building emergency shelters that are lightweight, easy to assemble, upgradeable, and cost-effective. This research aims to address gaps in existing modalities for emergency shelters during typhoon disasters through proposing an alternative design that uses CFS based on internationally accepted standards. The methodology is divided into three (3) phases, namely *(1)* analytical design and modeling, *(2)* cost sustainability assessment, and *(3)* shelter implementation plan. Three (3) different configurations with 4, 6, and 8 studs per wall face were designed and optimized using Midas Gen based on governing wind loads. The final frame design consisted of fully cross-braced along the failing direction, with an increase in uniform thickness of members. All of which checked for adequacy and connections designed accordingly. The overall cost was quantified lower than that of the traditionally used shelter design in terms of cost, covered area, and lifespan by a significant margin. All important information and findings are aligned in an original shelter implementation manual to ensure the shelter is aligned with the current situation in the Philippines.

## 1 Introduction

The Philippines is one of the most disaster-prone countries in the world. It is frequented by an average of 20 typhoons each year. Its destructive aftermath more prominently affects isolated remote locations; hence, relief operations are prolonged despite being time-bound [1]. However, available plans, modalities, and frameworks for typhoon response apply only to certain situations in most areas of the country and focus on short-term solutions and immediate survivability; thus, it does not consider other factors, such as improved living conditions and needs beyond shelter. During typhoon Yolanda in 2013, although available temporary shelters were provided to affected communities, the number of available tents was not able to support the large number of displaced residents [1]. Furthermore, ten months after the disaster, displaced families from Leyte and Eastern Samar still reside in temporary shelters provided by the United Nations, due to the government's failure to provide permanent housing. Moreover, it is said that millions of people still situate on makeshift shelters long after post-disaster. Post-disaster phases are typically divided into relief, recovery, and reconstruction [2]. This study primarily involves the relief phase, whose timeline is set immediately after a disaster. In the relief phase, basic survival needs are prioritized. Hence, this phase starts shortly after a disaster. Emergency housing is a form of necessary assistance provided upon initial disaster response, serving as a temporary shelter for the affected residents for protection following natural disasters [3]. Post-disaster shelters can be categorized into four phases: emergency, temporary, transitional, and permanent. Emergency shelter focuses primarily on the first few days of disaster aftermath [4]. The shelter aims to provide necessities such as refuge, physical safety, accessibility, and storage of essentials, such as water, food, and medical supplies. Moreover, a temporary shelter is a public mass shelter designed for habitability for a few weeks, while communities reestablish routines and wait for community restoration. Emergency shelters can also be utilized as temporary shelters should they be used for an extended period [5]. Hence, provisions for the structure's potential protracted usage must be integrated properly during its planning period. Transitional shelter, on the other hand, is expected to have a life span of six months to five years; as such, it is expected to be more robust in design and can provide an improved living condition given the timeframe. The last phase is through permanent shelter, which involves rebuilding houses. This phase is considered the costliest and most complicated phase as it involves a year-round commitment to be able to build back better and integrate potential measures that can alleviate risks of future disasters.

Shelter and housing recovery serve as one of the factors in overall recovery, as it instigates community restoration and relates to other community aspects such as health, dignity, and livelihood [4]. In the wake of certain disasters, rapid deployment of emergency shelters can provide support to displaced peoples in terms of health, safety, security, privacy, and protection as well as a sense of orientation and identity [6]. While proper implementation hastens the recovery period of the affected communities. It can minimize casualties and overall urban vulnerability to natural catastrophes [7]. Among the criteria in assessing post-disaster emergency shelters are: *(1)* quality, which encompasses aspects of structural, cultural and climate suitability, *(2)* ease of construction, and *(3)* cost-effectiveness [4]. An unstructured post-disaster response prompts residents to seek refuge in surrounding community structures, such as churches, public buildings, covered courts, which consequently may interfere with its original function. Currently, gaps in post-disaster planning instigate a lack of adaptive ideas for emergency shelters that respond not only to the basic shelter needs of the recipients but provide a more humane environment, which in turn affects the health, livelihood, and community function. Furthermore, the role of post-disaster shelter in human survival must extend beyond serving as a dwelling unit for the displaced population, as it contributes factors relating to safety and security, climate protection, and acts as a barrier from disease, hence, necessitates more than being habitable [5].

This study primarily focuses on designing an emergency shelter that addresses the need for a more economical and sustainable option. The goal is to provide convenience and efficiency through simple construction and a short inception-to-completion period, while also offering sufficient utility. It also proposes a design that provides ease of construction through a proposed implementation plan while being readily available shortly during a post-disaster response. According to the shelter recovery program milestones and targets of the Department of Human Settlements and Urban Development of the Republic of the Philippines and World Bank, the first regular communication with affected households occurs within 1 week of the disaster [8]. Consequently, this period allows the affected population to express their needs and lodge grievances. Hence, it is vital that within this period, necessities, which include a shelter with acceptable living conditions, is readily available. Furthermore, given the shelter's role in early post-disaster response, speed of construction and structural integrity are elements that are prioritized.

Considering the short timeframe for post-disaster response, cold-formed steel (CFS) framing was chosen as the primary material in this study. Cold-formed steel, is a type of steel formed by press-braking blanks sheared from sheets at ambient room temperature [9]. The term CFS has been used alternatively with metal studs, light gauge metal, light gauge steel (LGS) and lightweight. CFS framing first appeared in the early 20th century to simulate wood studs, the more common construction method for shelter at the time [10] CFS has been used in a variety of applications for different types of structures, more commonly mid to high-rise framing. Its main advantages include a high strength-to-weight ratio despite being lightweight, ease of assembly, and versatility in fabrication and configurability. Cold-formed steel framing in different configurations is expected to remain structurally sound even under higher stress and loading given its strong material properties. A key factor to consider when developing emergency shelters is the ease of use and on-site application, allowing residents to erect them without the need for professional supervision. Primarily, the emergency shelter is designed for ease of erection and deconstruction to support immediate post-disaster response. CFS members and shelter framing designed around the material can be distributed to communities and local government units in various ways as part of pre-disaster mitigation rollouts and post-disaster relief operations [11,12]. The study aimed to optimize cold-formed steel (CFS) framing for emergency shelters to withstand post-typhoon wind loads, utilizing Midas Gen software for detailed structural modeling to minimize deflections and ensure stability. It focused on specifying effective and practical connections for the shelter frame while evaluating the cost sustainability of CFS compared to traditional materials. The primary goal was to address gaps in current disaster shelter designs in the Philippines by providing a durable, cost-efficient, and versatile solution that enhances disaster response capabilities and ensures long-term utility. Additionally, in comparison to other materials used in current alternative temporary shelters, such as polyvinyl chloride (PVC) pipes, bamboo, and wood, the use of steel presents a possibility for shelter integration into permanent housing construction.

## 2 Review of related literature

### 2.1 Post-disaster response and recovery in the Philippines

In the latest iteration of the Post-Disaster Recovery Policy Framework by the Department of Human Settlements and Urban Development and World Bank (2022) [13] effective program, execution plan, and right deliverables. The government's Building Adequate, Livable, Affordable, and Inclusive (BALAI) Program aims to create resilient communities around the country as a response to the ever-changing climate and stronger typhoons hitting the country.

The Post-Disaster Recovery Framework Policy (PDRF) is prepared by the Department of Human Settlements and Urban Development (DHSUD) and the World.

Bank and contains most of the modalities on temporary shelters. The said framework focuses on the three phases of post-disaster response when providing structural housing or shelters. The housing or shelters provided may fall under emergency shelters, temporary housing or shelter, and permanent re-housing or relocation. Another category under transitional shelters may also be considered; however, this focuses more on the movability of the shelter and makes use of materials that may be reused for permanent housing. Under this category, there are five classifications depending on the situation: (1) Host Families (2) Self-settlement (3) Temporary Housing (4) Collective centers (5) IDP Camps.

The 3rd classification is the closest to the proposed prototype shelter. Normally, NGOs would take the initiative of coordinating with contractors to build bunkhouses and other structures needed. This, however, is avoided by the Post-Disaster Shelter Recovery Policy Framework (PDSF) due to the fact that conventionally building bunkhouses on-site take up funding that may otherwise be used for permanent relocation of displaced residents. Depending on the location, this may differ as it is expected that local government units adjust to the situation at hand.

To further improve emergency response on local levels, the MOVE UP Project was created. An Alternative Temporary Shelter System Manual [14] (MOVE UP Project, 2020) was developed specifically for LGUs to refer to whenever there is a need for immediate response to disasters. The manual was prepared based on universally accepted standards and is divided into 3 sections which are design, fabrication, and deployment. Alternative shelters differ for each community and location given differences in available resources, risks, land area, and capacity.

### 2.2 Cold-formed steel

According to Doctolero & Batikha (2018) [15], cold-formed steel (CFS) has seen multiple uses and applications in the past years from prefabricated framing to use for modular homes. It is a widely used building material with a variety of applications for different structures. Its versatility and usability are backed by its advantageous properties such as high strength-to-weight ratio, high stiffness, simplicity to prefabricate, easy erection, installation, and assembly, and recyclable and sustainable material as compared to wood.

Moreover, a study by Babu & Selvan (2021) [16] highlights both the advantages and potential future directives of CFS. Through the years, CFS has grown in usage as compared to hot-rolled sections. Its ability to produce high strength materials, while being lightweight, having shorter section spans, and a wide range of section configurations are among the reasons for its increased prominence in the industry. In addition, the Steel Framing Industry Association, mentions that cold-formed steel is not only considered a sustainable “green” material but can also be used in structural applications through the integration of panelized system technology.

A study explored cold-formed steel C-lipped wall studs subjected to major axis bending and performed 17 experiments with variation in the setups using the following [17].

• Oriented Strand Board (OSB)
• Plasterboard used as sheathing
• Varying connector spacing

According to the study, the quick and efficient way of constructing these walls make it the main option to use both as non-structural and load-bearing wall systems. Normally cold-formed steel members can be added for lateral bracing but adding sheathing can possibly increase bending and axial strength. The study focused on using Single-sided OSB sheathed studs and double-sided sheathed studs with OSB + gypsum boards. The main objective was to use closer-spaced connectors to improve the capacity of stud-wall systems.

### 2.3 Emergency shelter design considerations

Currently, guidelines on disaster relief (DR) shelter are being studied and continuously developed to understand its current context, especially on the aspect of environment, economic, technical, and socio-cultural. Disaster relief shelters are designed in a way where they can be erected, dismantled, stored, and reused. Hence, they are characterized as a lightweight structure that serves various purposes. More specifically the International Organization for Migration (2) [18] described DR shelters to have the capacity of being recycled, upgraded, reused, resold, and relocated after disassembly of unit. More so, upgradeability and reusability are factors highlighted as they highly contribute to pollution. Thus, it is suggested that shelters should be designed to be lightweight and have few materials in order for them to be easy to erect and dismantle in order for them to serve different functions.

The National Building Code (NBC) of the Philippines, as revised by Presidential Decree (PD) 1096, details the required ceiling heights, horizontal dimensions, and air space requirements for a habitable space. First, section 805 of the NBC entitled “Ceiling Heights” states that “habitable rooms” must have a minimum ceiling height of 2.40 m for artificially ventilated rooms or 2.70 m for naturally ventilated rooms. Next, section 806 of the NBC entitled “Size and Dimension of Rooms” states that “Rooms for Human Habitations” must have a total area of 6.00 square meters with a minimum smallest dimension of 1.50 m. Last, section 807 of the NBC entitled “Air Space Requirements in Determining the Size of Rooms” states that “Habitable rooms” must have a total air space volume of 14.00 cubic meters per person. The stated code provisions are for permanent residential dwellings, and the study will incorporate adjustments to account for the shorter duration of usage of temporary shelters.

### 2.4 Post disaster temporary shelters

MOVE UP Project (2020) considers standards to be followed in the setup of ATS at the site following the Camp Coordination and Camp Management (CCCM) Plan. The CCCM plan presents specific requirements for the type, number, and position of ATS units to ensure that internally displaced populations (IDPs) meet their needs in terms of safety, security, and privacy. The standards used for ATS based on CCCM include.

• A minimum area of 1.5 square meters for every person when considering safety and life preservation and a minimum area of 3.5 square meters for every person when considering the use of emergency or temporary shelters, or any area that is agreed to;
• A maximum of 50 m for the distance between latrines and temporary shelters;
• A minimum ratio of 1 latrine for every person;
• The presence of space for IDPs to store their assets; and
• The presence of important facilities for breastfeeding of infants, fostering children, and educating the general population.

The Transitional Shelter Guidelines by the International Organization for Migration (2012) [18] also presented the 5 characteristics of a transitional shelter, which are:

(1) Upgradable (2) Reusable (3) Relocatable (4) Resaleable (5) Recyclable. The upgradable transitional shelter should be open to improvements, more specifically as a permanent housing solution through maintenance, extension, or replacement to more durable materials. First, it must be upgradable where transitional shelter should be open to improvements, more specifically as a permanent housing solution through maintenance, extension, or replacement to more durable materials. It must also be reusable after the reconstruction phase, transitional shelters should be able to serve other alternative functions. Third, it must be relocatable, where this characteristic is definitive of a transitional shelter from other approaches. With this, a relocatable shelter can be built on temporary placements. It must also be resalable where upon dismantling, materials can be sold. Lastly, it must be recyclable as its dismantled materials can be reused for the construction of permanent housing. Given this, the researchers aim to be able to adopt the five aforementioned presented transitional shelter characteristics towards the proposed emergency shelter design in order to develop a more innovative approach and elevate the living conditions for emergency shelters.

### 2.5 Engineering software

midas Gen is a highly prevalent civil engineering software due to its user-friendliness and relatively simpler interface. It was used primarily in structural analysis and the design field of civil engineering. The application's features include a simple modeling interface, automated analysis and design, and quick generation of structural layouts, analyses, and reports. Its functionalities allowed different types of structural engineering applications, such as load combinations, member forces, capacities, displacements, and moments, among others. These results were easily integrated into a spreadsheet format for easier analysis of data. This software has been used specifically in studies such as structural deformation [19] and earthquake analysis [20].

## 3 Frameworks of the study

### 3.1 Conceptual framework

**Fig. 1:**
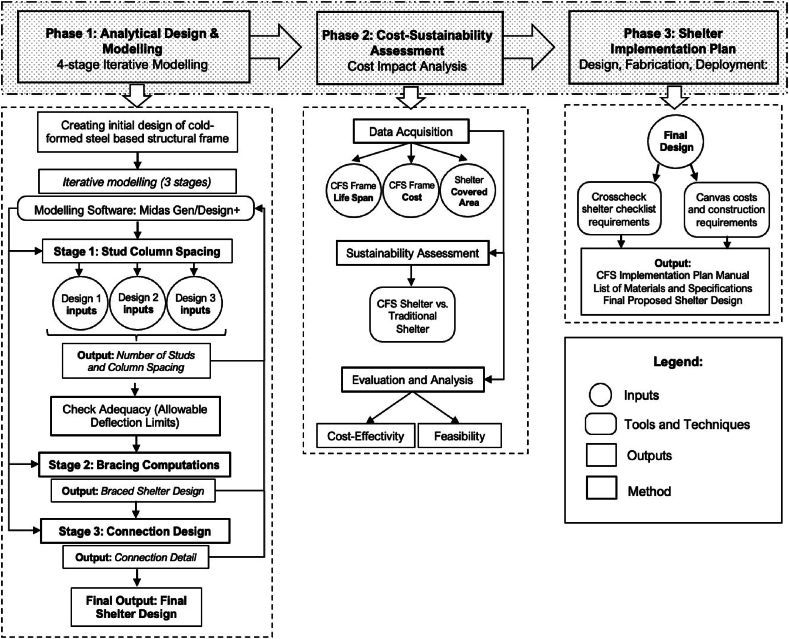
Conceptual framework of the study.

The design of a temporary shelter requires multiple considerations in piecing together a structurally sound frame whilst accounting for other aspects of construction that are not normally included in typical housing projects and shelters. A shelter frame built from components made purely of LGS necessitates following existing codes and references used by the industry. Fig. 1 shows the research methodology, divided into three main phases, each focusing on specific inputs, procedures, and outputs used to complete data collection and analysis.

The first phase is the Analytical Design and Modeling Phase. It is focused primarily on formulating the design of the shelter and performing structural modeling with predefined stages to assess its structural soundness in resisting loads. The initial shelter design, consisting of the frame components used, was based on multiple sources that detail the proper use of cold-formed steel for structural wall framing applications. The mentioned phase is divided into three stages, wherein each has a specified output for the aspect of the design considered. The stages for framing design include *(1)* modeling and analysis considering stud column spacing, *(2)* bracing computations, and *(3)* connection design. One input among those used in all three stages includes the static load cases to be considered in the structural serviceability requirements of the shelter frame, such as dead loads, due to the self-weight of members, theoretical roof live loads, and lateral loads due to wind pressure. Midas Gen was used as the structural modeling and analysis software for the first and second stages to obtain outputs of the final number of studs from stud column spacings, and maximum deflection experienced by every node in the model, respectively. The outputs of the first stage with the least maximum deflection were first used as the basis for the shelter frame, still without bracing, for use in the design of the bracings for the second stage. The collective outputs of the first and second stages were then used to select only one of the three shelter configurations, differentiated by the number of studs or stud spacing, for use in the third and last stage.

Following Phase 1, Phase 2 was conducted through a cost-sustainability assessment, where the shelter was assessed for its cost-effectiveness and feasibility. In this stage, a comparison between the proposed shelter design and use of a non-conventional material —CFS, was compared with currently implemented emergency shelters to assess whether the proposed emergency shelter is suitable as an alternative for implementation in the Philippines on the aspect of cost. Generally, given that the CFS material is compared to other accessible materials, such as plastic sheets, used in constructing emergency shelters, it is necessary to assess whether the application of CFS not only provides an advantage in terms of structural integrity but also for cost impact, specifically for long-term investment. However, due to the varying costs of factors such as labor and storage, as well as limited time and data, the researchers have decided to focus solely on the cost of materials. To perform the analysis, the cost in Php/m^2^/month, as utilized by similar studies [10], was obtained for the CFS-framed shelter and traditional emergency shelters. A lower cost (in Php/m^2^/month) is desired, as it will be indicative of cost efficiency.

Finally, Phase 3 contains the final output — a shelter kit manual patterned from Move Up Manual and the International Federation of Red Cross (IFRC) Shelter Kit, which contains all necessary information for the structural design, fabrication, and implementation of the proposed CFS framed shelter. Humanitarian relief and non-government organizations (NGOs) such as the IFRC and the International Organization for Migration (IOM), were also utilized as references in checking international requirements for a safe shelter. The content of the manual will include the final framing design, a list of framing materials, instructions for assembly and disassembly, proper storage, and options for other shelter components, such as foundation, covering, serving as a reference guide for stakeholders in the proper implementation of the emergency shelter that is necessary for immediate disaster response.

### 3.2 Theoretical framework

**Fig. 2:**
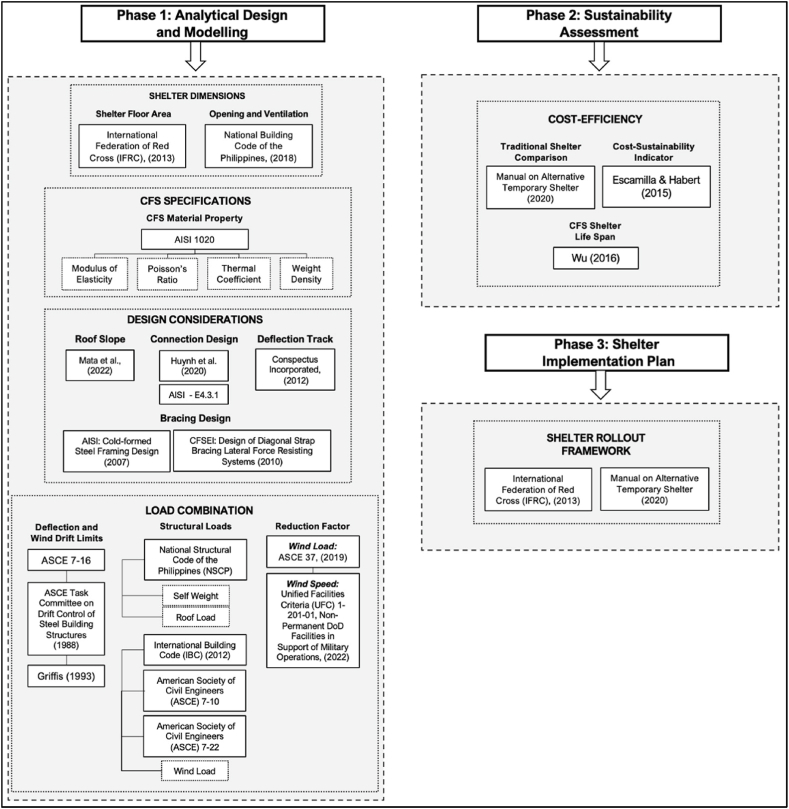
Theoretical framework of study.

The study primarily focuses on three stages, namely Phase 1: Analytical Design and Modelling, Phase 2: Sustainability Assessment, and Phase 3: Shelter Implementation Plan. Fig. 2 summarizes the theoretical framework of all phases in the study, which indicates all reference literature and standards adopted [[21], [22], [23], [24], [25], [26], [27], [28], [29], [30], [31],[35], [36], [37], [38], [39]].

Phase 1 of the modeling process focuses on an iterative process of designing the CFS-framed emergency shelter; hence, it must be adherent to structural requirements in order to meet acceptable design criteria. Given this, various provisions from existing structural codes, standards, and studies are adopted during the design process to ensure structural integrity. The framework for Phase 1 is divided into four, namely: shelter dimensions, CFS specifications, design considerations, and load combinations. The shelter dimensions were patterned from the International Federation of Red Cross and Red Crescent Societies [22], specifically from an existing temporary shelter design. Moreover, sizes for opening and ventilation follow the guidelines from the National Building Code of the Philippines [23]. Moreover, given that CFS was the primary material applied for framing and bracing design, it is necessary to identify its specifications to properly integrate into the design process. The material properties, specifically the modulus of elasticity, Poisson's ratio, thermal coefficient, and weight density were necessary inputs for Midas Gen to properly simulate the use of CFS as a material in structural modeling. These properties were adopted from the American Iron and Steel Institute (AISI) [32], which contains the specifications for cold-rolled steel. On the other hand, the design consideration adopted existing studies, provisions, and design principles for the modeling process of the shelter [24]. Similarly, the connection design for steel members was adopted from previous studies [25], and specifications of AISI - E4.3.1. Deflection tracks used to support potential deflection on the roof and floor assembly adopt provisions from Conspectus Incorporated. For bracing design, the type of bracing applied was based on a recommendation from Cold-formed Steel Framing Design of AISI. Meanwhile, the computation for bracing cross section was based on Design of Diagonal Strap Bracing Lateral Force Resisting Systems by the Cold-Formed Steel Engineering Institute (CFSEI) [26]. Lastly, load combinations were applied to identify the shelter's limit toward loads acting on the structure. Provisions for structural deflection and wind drift limit, structural loads applied, such as self-weight, roof load, and wind load, and respective reduction factors relating to the shelter type were also integrated to account for the design limits and provide realistic parameters for the shelter type.

As for Phase 2, to solve for the cost-sustainability assessment adopted from existing studies, specifically on the average CFS shelter life span, and the methodology to compute the cost-sustainability, existing emergency shelter designs from the Manual on Alternative Temporary Shelter were also used as bases of comparison for the proposed CFS shelter in terms of cost-efficiency [31]. The materials list and lifespans of the existing shelters were sourced from the said manual. The last stage mainly focuses on creating a shelter rollout manual, or the shelter kit. The IFRC [22] and Manual on Alternative Temporary Shelter [31] are two references that tackle existing shelters and their application, and shelter specifications, such as background, cost, life span, materials, performance analysis, and potential upgrade. Hence, they mainly focus on creating a reference for stakeholders that will help improve the post-disaster shelter conditions, where the content focuses on helping these stakeholders grasp a deeper understanding of the proposed shelter solutions. With this, Phase 3 aims to adopt applicable suggestions and guidelines from these manuals and improve them by integrating the proposed design of the CFS-based shelter.

## 4 Research design and methodology

**Fig. 3:**
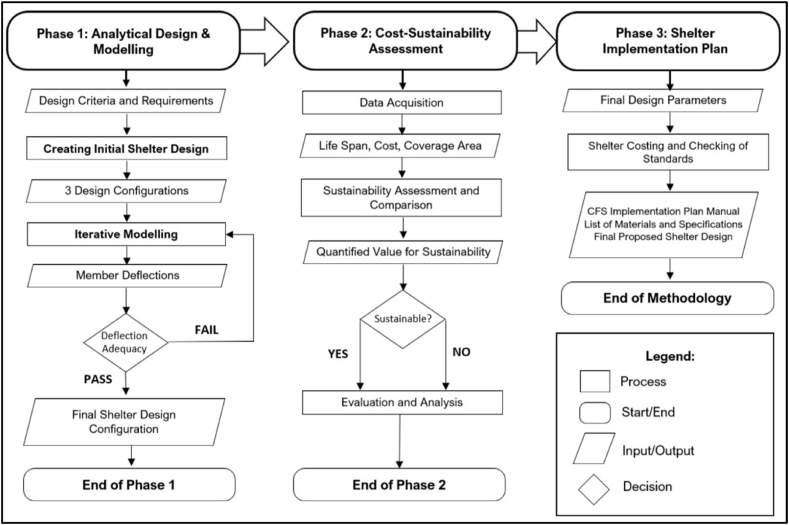
Research methodology flowchart.

The study used a quantitative design to develop an emergency shelter framing design utilizing cold-formed steel members. The analytical design was based on various sources and references specific to CFS construction considering quantifiable measurements and dimensions for all structural components. Through incorporating existing values in the initial design of the structural frame, shelter construction was limited to both internationally accepted standards and the certain assumptions specified in this study. Relationships between multiple variables with corresponding results in the modeling software used established parameters to determine whether the design is structurally viable and economical. Additionally, the obtained results were used in conjunction with the formulation of a shelter implementation plan manual which is a qualitative output that serves to summarize and actualize the final plans. A summarized flowchart of the overall step-by-step procedure based on the conceptual framework of the study is shown in Fig. 3.

### 4.1 Phase 1 – analytical design

#### 4.1.1 Floor area and dimensions

According to The Sphere Handbook: Humanitarian Charter and Minimum Standards in Humanitarian Response [33], one key indicator in designing a shelter is the adequate living space and the ability of those using it to carry out daily activities. Moreover, an adequate floor area must be supported by a building enclosure, including walls, doors, windows, and roof, in order to create a dignified environment. Additionally, the time, location, and overall severity of the conditions must be accounted for; therefore, in choosing the floor area, the aspects of simplicity and ease of assembly were prioritized, limiting the number of materials used for a shelter that aims to be immediately available within 1–2 days of the disaster occurring. Unlike previous shelters that include multiple rooms, the shelter design made in this study is focused mainly on rapid construction, to immediately serve being set up as fast as possible and serve the affected community by providing a covered area that is structurally sound and safe to stay in. A 3m × 3m inside area was chosen akin to the design of barrel vault tents for outdoor settlements included in the Manual for Alternative Temporary Shelters by MOVEUP [30].

Furthermore, approximately 5–7 family members can comfortably fit within the 9 square meter area coverage [31]; however, other literatures still recommend a 3.5 square meter per person area for better living conditions, excluding the area for bathrooms and other areas [33]. According to the Philippine Statistics Authority, the average household size in the Philippines is 4.1. With this, 3m × 3m was deemed acceptable for a shelter built for immediate response in providing adequate living space as compared to other shelters that aim to maximize the ability of residents to conduct daily activities for a prolonged period while waiting for permanent rehousing and resettlement.

**Fig. 4:**
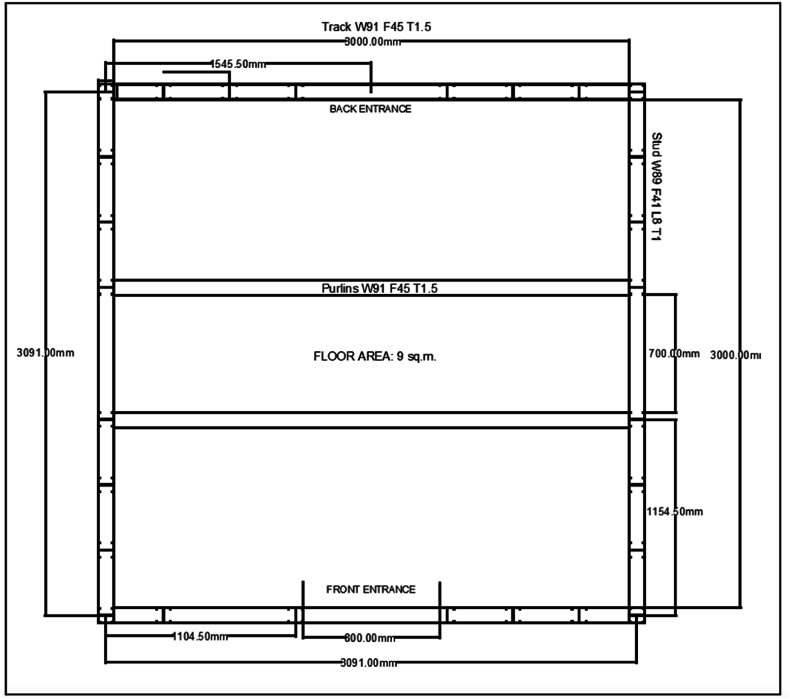
Shelter livable floor area.

The proposed shelter's intended use will primarily be for outdoors, as the target for the proposed design are areas that do not have immediate access to nearby buildings that can serve as evacuation centers. Given that it will primarily be utilized only for a shorter period, ideally while waiting for the construction of bigger transitional shelters, approximately 5–7 people sitting down can utilize the space inside. However, this will not provide the most comfortable conditions for those affected but mainly focuses on simply providing coverage from the outdoor environment. As such, it is recommended that a maximum of 2–3 people per shelter are to be accommodated if the shelter is used for a longer period of time, as this will allow for more space, not just for lying down but to accommodate more movement inside. The said recommendation is in line with the recommendations of The Sphere Handbook [23]. The floor area measurements of the design are shown in Fig. 4. Lastly, an internal floor-to-ceiling height of 2.4 m was applied to the structure [33].

#### 4.1.2 Openings and ventilation

According to the Rule VII entitled Light and Ventilation of the National Building Code of the Philippines, habitable rooms intended for use that do not have any artificial or man-made ventilation systems should have window openings with a total area of 10 % of the floor area at the very least [23]. As previously discussed, the floor area coverage of the shelter was at 9 square meters, which necessitates at least 0.9 square meters in total for window openings. To provide better air circulation and lighting during the day, the shelter was designed to have 2 windows, measuring 0.7 m × 0.7 m, on each side, conforming to the NBC requirement. Other considerations for specific instances, where open shelters cannot be used in the area, are discussed with probable options in the shelter implementation plan.

As for the doors, there is 1 on each of the opposite sides of the frame that does not have a window. This provides easier access in and out of the shelter and helps with the overall ventilation inside. Additionally, it provides an avenue for possible expansion of floor area connections in cases where more than one of the same shelters is to be connected side by side.

#### 4.1.3 Main structural components

The main structural components to form the entire frame consisted of only cold-formed steel studs and tracks. For its cross-sectional properties and dimensions, a local company in the Philippines was consulted to obtain readily available specifications that are currently being fabricated in the industry. This was done to limit the useable members for the design and ensure that the proposed frame is and will be viable to produce. Among the available options, the smallest profile for studs and tracks was used, which has a corresponding web measurement of 89 mm for the studs, and 91 mm for the tracks, and a flange width of 41 mm and 50 mm, respectively.

It is impractical to mix different thicknesses of studs and tracks in the same project, as this would only increase confusion and costs in actual construction and must be done only when necessary for structural requirements [34]. Because of this, the jamb studs considered in the design made use of the same stud profile that was used for the columns, placed along all sides of the base. According to AISI [34], multiple ways exist to connect and combine studs to form built-up members. Jamb studs are built-up members that need to be placed immediately adjacent to an opening in the structure to provide additional support. While studs can either be connected back-to-back or toe-to-toe, the said standard states that a toe-to-toe configuration is not recommended when predominantly using screws to connect studs together due to the difficulty of attachment and disassembly as opposed to when the members are welded together. With this, a Jamb stud of the back-to-back configuration was used for all openings in the frame.

Typically, partitioning systems are usually constructed not to be load-bearing; however, in cases where wall systems are required or expected to carry the load, framing assemblies are designed considering deflection and bending due to resisting dead and live loads. For the shelter's base, it was expected that the tracks used would be wider than the stud columns as it forms the perimeter of the shelter where the studs will be attached. The same type of track was then used to secure the stud columns, and jamb studs on top to serve as the connection between the column members and the roof truss and was designed to account for the deflection which may be induced by the dead loads due to the material's self-weight and live load.

#### 4.1.4 Roof design

The type of roof was chosen based on characteristics necessary for a temporary shelter, including quick and easy assembly and high-cost efficiency. Moreover, oftentimes, the complexity of a shelter's structural design leads to delays in construction, the worst being completed after the rebuilding of permanent homes [22]. With this, there was a necessity to consider timeliness and construction speed must be considered to address immediate needs. Hence, the proposed design used a skillion roof, characterized by a singular slope, and is amongst the simplest types of roofs. In addition, a slope in the roofing is necessary to accommodate rainwater runoff and avoid ponding. Other researchers found that the optimum roof angle for single-family residential houses in the Philippines was 11° using a methodology involving genetic algorithm (GA) in MATLAB and computational fluid dynamics (CFS) in Solidworks [24]. As such, a skillion roof with a 11° initial roof angle was adopted in the roof design of the temporary shelter.

#### 4.1.5 Stud and built-up jamb stud spacing

In every light gauge steel frame construction, an important parameter that affects the overall strength and stability of the structure is the on-center spacing of the studs. This determines how many studs will be placed along one side or face of the wall. One of the main objectives of designing a shelter for immediate response is to lessen the amount of material used as much as possible to further the ease of assembly and lower the overall costs from manufacturing until actual use in disaster-struck locations. With this, the structural analysis in terms of maximum displacement and experienced beam stresses of 3 different types of configurations with varying numbers of studs was done to identify whether or not a specific number of studs will significantly perform better for a small coverage area of 9 sq.m. To ensure that results will vary only with the number of studs per configuration, all other parameters, such as member thickness, floor height, roof pitch, and applied loading cases, were kept constant. In modeling each of the 3 configurations, the center-to-center spacings of the member sections were used to ensure uniformity and avoid any unwarranted changes in the orientation and spacing of any component of the shelter.

Typical spacings of 8 inches (203.2 mm), 16 inches (406.4 mm), and 24 inches (609.6 mm) were used for each model configuration to compute and determine the number of studs that can fit using the given on-center spacings. After obtaining this number, all the studs were equally spaced along the length of the corresponding side. This is to ensure that the number of studs remains the dependent variable in the comparison of all 3 configurations. It must be noted that jamb studs are present immediately at the edges of the openings which were not considered in the computation of center to center of spacing of the single studs.

#### 4.1.6 Wind load profile and parameters

*A*SCE 7–16 and ASCE 7–22: Minimum Design Loads and Associated Criteria for Buildings and Other Structures is the main reference in the manual calculation verification of wind loads via the directional procedure using the general basic parameters required in determining wind loads for a Main Wind Force Resisting System (MWFRS) and the allowable drift limits which was set to be 1/400 of the shelter's height.

The final parameters based on the NSCP Section 207 used for the wind loads acting on the final shelter model is shown in Table 1:

**Table 1:** Wind load parameters.

Occupancy Category	IV
Wind Directionality Factor, kd	0.85 (MWFRS)
Exposure Category	C
Gust Effect Factor	0.85
Building Classification	Low rise, partially enclosed
Internal Pressure Coefficient (GCpi)	+-0.55
Wind Speed (kph)	156

The emergency shelter is identified as a category IV structure not falling within categories I, II, and III, which are occupancy classifications for treatment areas, buildings with a capacity of 1000+ and hazardous facilities. Occupancy category V on the other hand is for miscellaneous structures, which are assumed to be for structures that are supposedly unoccupied. The wind directionality accounted for in the study is Kd = 0.85, as it is considered a solid freestanding structure given that the shelter does not fall within any other category. The structure is identified as having exposure category C, given that B and D are not applicable due to the requirements of the ground surface roughness while the gust effect factor is taken as 0.85 based on the standards of the code provisions.

#### 4.1.7 General modeling process

The general modeling process, using Midas Gen and Midas Design+, started with defining the material properties of the structural members to be used for the temporary shelter. Table 2 presents the material properties defined in the model for CFS [32].

**Table 2:** Summary of material properties used.

Parameter	Value
Modulus of Elasticity	186 GPa
Poisson's Ratio	0.29
Thermal Coefficient	7.72 μin/in-°F
Weight Density	77.18 kN/m^3^

After defining the material properties, the process was continued by defining the section properties of the structural members to be used for the temporary shelter. The dimensions of cold-formed steel studs and tracks from a local company were used to define the sectional properties in the model. Table 3 presents the four types of sections used in the model based on the available dimensions of the local company.

**Table 3:** Summary of section properties used.

Description	Orientation	Description
W89 Studs	Vertical	Single Cold-Formed Steel Studs
W91 Tracks	Horizontal	Single Cold-Formed Steel Tracks
W89 Built-Up Jamb	Vertical	Double Cold-Formed Steel Studs Connected Back-to-Back
W91 Combined Track	Horizontal	Double Cold-Formed Steel Tracks Connected Back-to-Back

In this study, the configuration considered for the iterative modeling process incorporated the number of studs as a basis and referencing the standard on-center spacings of 12, 16, and 24 inches from varying configurations. Since minimal differences were found after adding stud spacings of 8 and 20 inches, the number of studs is calculated from the provided standard stud spacings. The studs were then equally spaced between the corner stud column and the jamb stud, resulting in an actual spacing of a little less than the specified standard stud spacings. To simplify the relationship of the configuration naming, the total number of studs between two corner stud columns on one side of the shelter was used instead of between one corner stud column and one jamb stud. The final set of configurations involved adding diagonal strap bracings, supplementing diagonal straps to produce full cross bracings, and increasing uniform member thicknesses to ensure the serviceability of the structure in terms of node deflection under the applied loads.

**Fig. 5:**
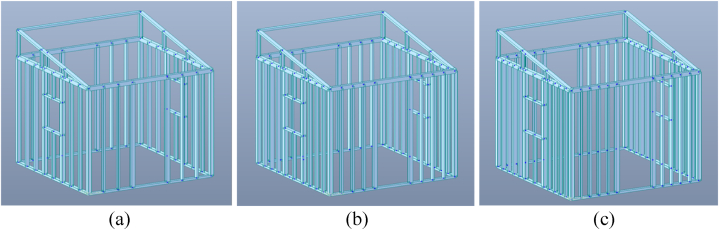
Final stud configuration geometry, with material and section properties, considering *(a)* 4 studs, *(b)* 6 studs, and *(c)* 8 studs.

After creating the geometry, material and section properties were assigned to the elements. For material properties, all elements were assigned using the material data of cold-formed steel. For section properties, on the other hand, each element was assigned the section data for the corresponding type of section. Fig. 5(a), (b) and 5(c) show the geometries of the final 4 stud, 6 stud, and 8 stud temporary shelter configurations, respectively, with the material and section properties incorporated.

The modeling process was then continued with defining and adding all the relevant loads for the study, which included self-weight, roof live load, and wind load. There was a total of 12 static load cases considered, with 10 wind load cases. Each of the static load cases for wind load was characterized with different orthogonal direction factors to consider the worst loading combinations of wind directions possible.

The reduction factors were adopted based on the guidelines of the American Society of Civil Engineers (ASCE) [40], where reduction factors were applied to wind speeds for structures with a construction period of less than six weeks. Given that the proposed study centers on emergency shelter that aims to be readily available within 1–2 days post-disaster, a 0.75 reduction factor for wind load was applied.

#### 4.1.8 Adequacy check for deflection and wind drift limits

The drift of walls and frames was computed using a building height of 3 m, giving an allowable drift of 0.005 m–0.0075 m for the deflections. For this study, 0.0075 m was the value taken as the limit of deflections along x and y for all 3 models. Each of the 3 will go through an iterative process of optimization, such that there will be 4 stages where the displacements on the nodes were checked for adequacy. In cases where nodes go beyond the minimum allowable drift of 0.0075 m, the models will move on to the next stage where a certain component was designed accordingly to increase the resistance to loads. For this study, the first stage consisted of modeling the 4, 6 and 8-stud configurations without any type of bracing, diagonal member for the roof truss, or purlins. Should the displacements of the nodes along either the x or y-direction still go beyond the limit, the second stage was continued, wherein a bracing was designed to be connected from the corners of the structure to the bottom edge of the jamb stud immediately adjacent to the opening. In the event that this design would still not be adequate, a full cross bracing was added along the side of the wall parallel to the direction where node displacement fails. The last stage of the model consisted of changing the uniform thickness of all the members.

#### 4.1.9 Connection design

In designing the connections of the proposed shelter, AISI and American Society for Testing and Materials (ASTM) Standards were used [[49], [50], [51]]. This includes establishing the material parameters, such as grade of steel, minimum yield strength, maximum tensile strength, as well as the specifications of fasteners. Similarly, AISI Standards were used in determining the minimum spacing, minimum edge and end distance, shear limit state, tension limit state, combined shear and pull-over, and rupture limit state of the connections.

### 4.2 Phase 2 – cost sustainability assessment

Cold-Formed Steel is a relatively a more expensive alternative to traditional materials for emergency shelters but is also more durable. Cost efficiency is a vital factor in the implementation of the CFS shelter. To assess the trade-off of cost for longevity, a sustainability assessment focusing on cost was performed, comparing traditional emergency shelters and the modeled cold-formed steel emergency shelter. The methodology for undertaking the said assessment was obtained from a study that studied the sustainability of different traditional shelter designs [21].

The Cost Sustainability Assessment in this study included two (2) phases. Phase 1 covers data acquisition to obtain the necessary data inputs for the assessment, including lifespan of the shelter, cost of the frames, and shelter floor area. Phase 2 pertains to the sustainability assessment and includes the cost assessment for the CFS shelter and traditional shelter. Material cost is a vital component in this assessment, as the goal is to evaluate the cost of the shelter given its factors. It should be noted that only the frames were compared with each other to simplify the comparison and that other components, such as the foundation, covering material, and facilities, were not included. A shelter area is a factor that represents the usability of the structure, as it defines the amount of useful space that its users can inhabit. Lastly, lifespan is an important factor since it describes the length of time the shelters can remain in effective functionality. Furthermore, it is inversely related to cost since the longer the product's lifespan, the less times it will need to be replaced. For this study, the lifespan considered will not just be for the period of shelter deployment and erection, but for the complete lifespan, essentially considering the reusability of the shelters. In view of this, the assessment was computed on a material cost per shelter area, per lifespan (Php/m^2^/month).

As mentioned earlier, the cost sustainability required three factors: total frame cost (in Philippine Peso), shelter covered area (in m^2^), and lifespan (in months). The total frame cost of the proposed shelter was based on the price provided by a local supplier of cold-formed steel in the Philippines.

Moreover, the lifespan of cold-formed steel frame structures was identified to be 30–50 years [41], with the potential of further usage after deconstruction and recycling at the end of service life. For a conservative estimate, this study utilized 360 months (30 years) as the estimated lifespan of the proposed cold-formed steel emergency shelter.

The traditional shelters that were compared with the proposed CFS shelter were obtained from the MOVE UP Manual [31], as they contain shelters designed for emergency use in the Philippines. From the said manual, shelters that show deployments and trial setups in the Philippines were selected, as it indicates the shelters had prior usage or are the most potentially likely designs for deployment in times of need. Through these criteria, four shelter designs were selected — PVC, GI Pipe, coco lumber, and bamboo.

The same manual lists all the materials required to construct a specific shelter. From this, the framing materials were differentiated, and the costings were based on available data in the country [42,43]. All prices were adjusted to the year 2023 to account for the inflation rates. For the floor areas, most of the shelter designs, including the PVC, GI Pipe, and bamboo, followed a size of 9 m^2^. The coco lumber shelter was found to have an area of 23.04 m^2^. Lastly, the lifespans were obtained from MOVE UP Project [21], which states that their lifespans have an average of at most 5 years. Due to the shorter lifespan of coco lumber and bamboo, their lifespans were obtained from Global Shelter Cluster [44] and Kaminski et al. [45].

### 4.3 Phase 3 – shelter implementation plan

The shelter implementation plan primarily aims to adopt current shelter rollout frameworks, specifically from the MOVE UP Alternative Temporary Shelter (ATS) Manual and the International Federation of Red Cross (IFRC). Excerpts on the content and presentation of data from both references were integrated into the proposed shelter rollout for the CFS-based shelter to support users and provide guidance regarding a newly proposed emergency shelter approach. Commentary on potential improvements in specific shelter components was also presented for further performance analysis. Moreover, this study covers discussions on proper storage conditions and methodologies [[46], [47], [48]].

## 5 Results and discussions

### 5.1 Phase 1 – analytical design

#### 5.1.1 Iterative Modeling Stages and Member Deflections

After a series of iterative modeling, and considering various load combinations, the final frame design consisted of fully cross-braced along the failing direction with an increase in uniform thickness of members. Table 4 summarizes the parameters for all iterative modeling stages, and details the number of diagonal bracing straps, uniform member thickness, and roof angle.

**Table 4:** Summary of parameters for all iterative modeling stages.

Stage	Name	Bracing (x-direction)	Bracing (y-direction)	Uniform Member thickness (mm)	Roof Angle (degrees)
1	Initial Design	None	None	1	11
2	1 Diagonal strap bracing, purlins and roof truss diagonal members	4 Diagonal Straps	4 Diagonal Straps	1	11
3	Fully Cross-braced along failing direction (x-direction)	8 Diagonal Straps	4 Diagonal Straps	1	11
4	1 Diagonal strap bracing, purlins and roof truss diagonal members	4 Diagonal Straps	4 Diagonal Straps	1.5	11
	Fully Cross-braced along failing direction and increased uniform thickness	8 Diagonal Straps	4 Diagonal Straps	1.5	11
	Fully Cross-braced along failing direction and increased uniform thickness	8 Diagonal Straps	4 Diagonal Straps	1.5	10
	Fully Cross-braced along failing direction and increased uniform thickness	8 Diagonal Straps	4 Diagonal Straps	1.5	9

**Fig. 6:**
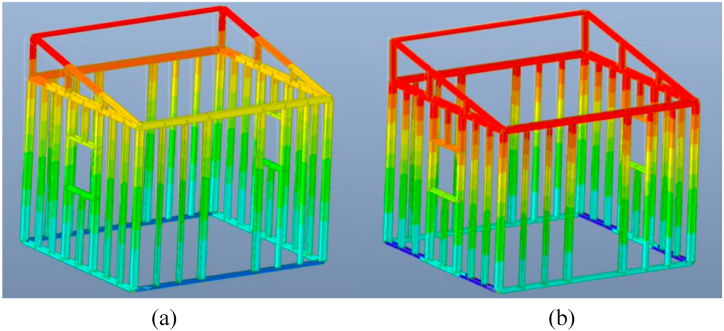
Displacement contour along the *(a)* x direction and *(b)* y direction using Midas Gen considering Stage 1 Modeling of 4 Stud Configuration.

The initial shelter design only comprised of 1 mm uniform member thickness, with 11 degrees of roof angle. No bracing was considered along both x and y directions. The said configuration did not pass the deflection limit of 0.0075 m. Fig. 6(a) and (b) show the contours of the shelter that represent the highest deflection along the x and y directions, respectively, relative to other nodes using Midas Gen. This was achieved considering the 4-stud configuration. The red portion in the figure corresponds to critical members based on deflection.

**Fig. 7:**
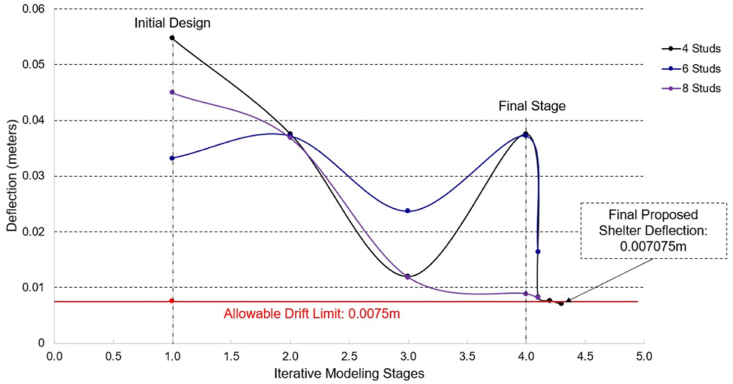
Maximum deflection values of all iterative modeling stages along x-direction.

**Fig. 8:**
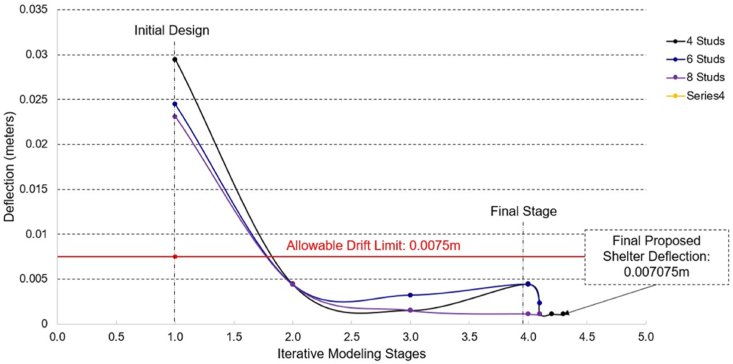
Maximum deflection values of all iterative modeling stages along y-direction.

As the iterative stages progressed, from Stage 2–3, the number of diagonal straps was increased from 3 to 4, and the uniform member thickness was increased from 1.0 mm to 1.5 mm for all components. It is within stage 4 where the roof angles are further decreased to lessen the excessive movement at the top of the roof truss. The progression of maximum deflections through the stages along the x and y directions are found in Fig. 7, Fig. 8, respectively.

**Fig. 9:**
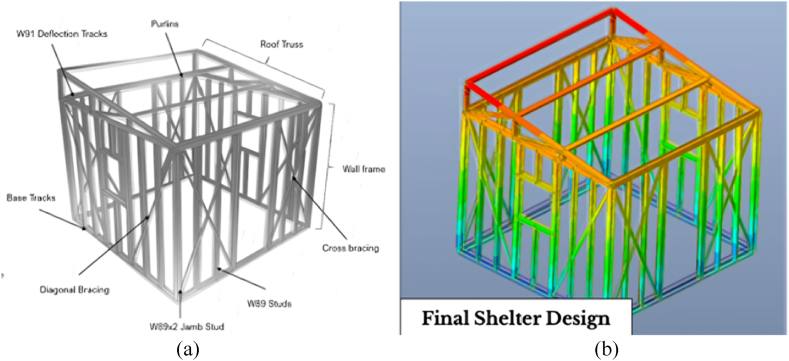
Final shelter design, showing *(a)* all structural components, and *(b)* the structural model generated in Midas Gen.

All node deflections using the 4-stud configuration, with 1.5 mm member thickness and 9-degree roof angle, remain within the 0.0075 m limit for deflection, thus the said configuration was considered the final design of the iterative modeling process. Fig. 9(a) shows the different components of the final shelter design. Correspondingly, Fig. 9(b) shows the structural model generated from Midas Gen, along with the contours of the shelter that represent the highest deflection values relative to other nodes.

#### 5.1.2 Connection design

**Fig. 10:**
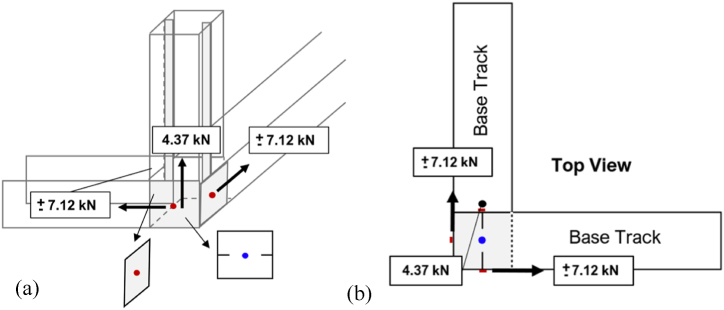
Corner connection member forces: isometric view (a), and top view (b).

In terms of the corner connections, four groups of connections per node were considered to resist the applied loads, as obtained from Midas Gen software based on the four bottom corner connections. Three groups of the said connections were located on three sides of the stud column, connecting track flanges to a column stud web. On the other hand, the last group of the said connections was located on the bottom, for the tracks, or top, for the deflection tracks, of the stud column, connecting the two webs of the intersecting tracks. The first group of three connections was taken to resist the applied load, while the last group of connections was designed to individually resist the applied load apart from the first group. This is because of the non-uniformity of the connections, such that the two intersecting tracks are only connected through their webs while the stud column is only connected to the flanges of the tracks. Additionally, the bottom group of connections were taken separately from the other connections, as they were designed to transfer the loads of the structure to the foundation. Fig. 10(a) and (b) illustrate the isometric view and top view of the member forces acting on the corner's groups of connections, respectively.

Each node shown in Fig. 10 represents the calculated number of screws required. On the other hand, the three red nodes represent the three groups of connections to act as one while the blue node represents the one group of connections to act on its own. The connections for the corners were also designed to be attached as pinned connections to the foundation of the shelter because there is no perfectly fixed condition in actual construction. Additionally, the load resisted by the corner connections was assumed to be the reactions at the four pinned supports of the shelter.

For all the other connections, only two groups of connection per node were considered to resist the applied loads as obtained from Midas Gen. The two groups of connections were located on either web of the studs and tracks, connecting the flanges to each other. Following the capabilities of the Midas Gen, the calculated member forces were only provided in the local axis of each member. Since reorienting the member forces to the global axis would be a tedious and inefficient process, a conservative estimate of the summation of the magnitudes of member forces along the local axis was used to design the connections.

**Fig. 11:**
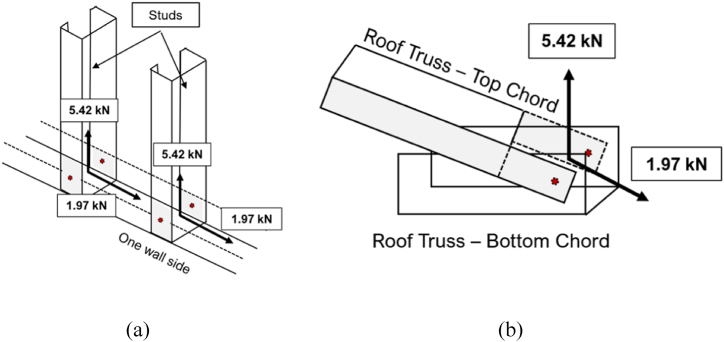
Other connection member forces: *(a)* Stud and Track, and *(b)* Roof Truss Top and Bottom Chords.

Fig. 11(a) shows the standard connection of studs to tracks. Like the corner connection, each node represents the total calculated number of required screws. In the analysis, the stud-to-track connection was assumed to be representative of all other connections, excluding corner connections. One such example of connections represented is also shown in Fig. 11(b), connecting the roof frame top chord to the roof frame bottom chord.

Considering both axial and shear forces, the final nominal diameter of the screw was obtained by iteration based on the governing limit state, particularly the tension pull-out limit state for the corner connections. Moreover, the final diameter that governed was also used for the other connections to simplify the needed screws for the shelter to only one type. The required inputs and calculated outputs for each limit state, which includes shear limit state, tension limit state, combined shear and pull-over, and rupture limit state.

**Fig. 12:**
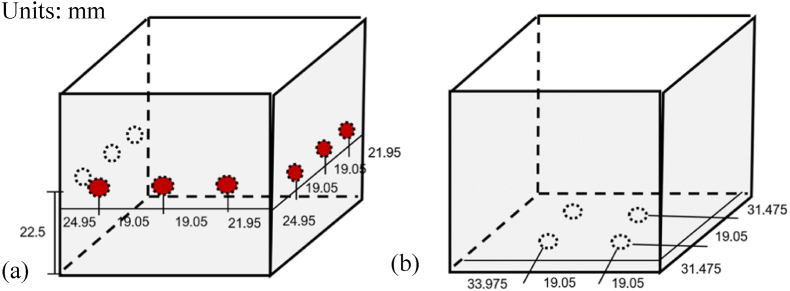
Final corner connections of Stud Column Sides, and *(a)* Track Flanges and *(b)* Track Webs.

**Fig. 13:**
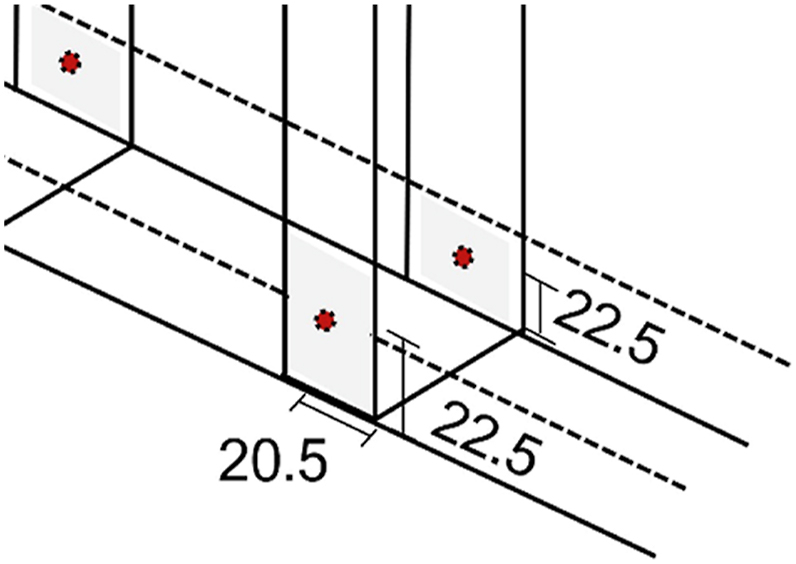
Other connections, as finalized.

In terms of the final connections, the on-center spacings were assigned to be 19.05 mm, based on the minimum requirement of 3 times the nominal screw diameter. The edge and end distances were rechecked to meet the minimum requirements of 9.525 mm, based on 1.5 times the nominal screw diameter, and 12.96 mm, based on the performed calculations for end distance, respectively [[49], [50], [51]]. The final corner connections, specifically for the different connections, are shown in Fig. 12, Fig. 13. In particular, Fig. 12(a) and (b) show the corner connections of track flanges and track webs onto the stud column sides, respectively, while Fig. 13 illustrates the other connections, as finalized.

All other connections, aside from the corner connections, were assumed to follow the standard stud-to-track connection shown in Fig. 13. Overall, the calculations yielded a minimum requirement of 13 screws per corner connection node and 2 screws for all other connection nodes. Since the shelter has a total of 86 connection nodes and 8 are corner connection nodes, the remaining 78 connection nodes were categorized under other connection nodes. Considering these parameters, the total number of screws was found to be 260 screws. The said calculation was based on conservative estimated values of forces acting on the nodes of the structure; thus, it is possible to further increase the efficiency of the connection design in terms of structural stability and cost by considering the actual forces. Nonetheless, the estimated value of screw connections required were obtained mainly for the cost-sustainability assessment of the shelter such that the stability of the structure and safety of the occupants are met.

### 5.2 Phase 2 – cost sustainability assessment

As previously discussed, the cost sustainability of the proposed design was obtained by using the inputs of cost, floor area, and lifespan in Php/m^2^/month. The result was compared with other emergency shelter design framing [31]. The shelter cost was limited to their framing components. Table 5 presents the Bill of Quantities, summarizing the total materials required as well as their cost. The price used for steel was Php 115 per kg.

**Table 5:** Cold-formed steel framed shelter costing.

Structural Member		Member Length (m)	Member Thickness (m)	Volume Per Piece (m^3^)	Mass Per Piece (kg)	Quantity (piece)	Price Per Piece (Php)	Total Price (Php)
Column (Stud)	2.3775	0.0015	0.0006	5.0671	16	582.72	9323.48	
	1.0550		0.0003	2.2485	2	258.58	517.15	
	0.5775		0.0002	1.2308	2	141.54	283.09	
Jamb (Stud)	2.3775		0.0006	5.0671	12	582.72	13,985.21	
Roof Truss Web (Stud)	0.4900		0.0001	1.0443	2	120.10	240.19	
	1.0800		0.0003	2.3018	2	264.70	529.41	
	0.3300		0.0001	0.7033	2	80.88	161.76	
	1.0400		0.0003	2.2165	2	254.90	509.80	
	0.1600		0.00004	0.3410	2	39.22	78.43	
Base (Track)	3.0910		0.0008	6.4786	4	745.04	2980.15	
Roof Truss Chord (Track)	3.1300		0.0008	6.5603	2	754.44	1508.87	
Roof Purlin (Track)	3.0910		0.0008	6.4786	3	745.04	2235.11	
Window Member (Track)	0.7820		0.0002	1.6390	4	188.49	753.96	
Deflection Member (Track)	3.0910		0.0017	12.9572	4	1490.07	5960.29	
Bracing (Window Side)	2.6500	0.005	0.0006	4.9406	8	568.17	4545.35	
Bracing (Door Side)	2.6300		0.0006	4.9033	16	563.88	9022.08	
Screws		*n/a*				260	8.25	2144.19
**TOTAL:**								**54,778.53**

Furthermore, the framing costs for the traditional shelter designs, namely PVC, coco lumber, GI Pipe shelters, and bamboo are summarized in Table 6, Table 7, Table 8, Table 9 respectively.

**Table 6:** Shelter framing cost using PVC.

Material	Quantity	Unit Price (Php)	Price (Php)
1 1/2″ PVC Pipe	18	277.01	4986.15
PVC pipe elbow 90	10	50.00	500
PVC Pipe tee	34	74.00	2516
Duct tape	1	265.55	265.55
20 M Nylon Rope	1	82.11	82.11
1 1.2″ metal type tk screws	88	36.30	3194.4
**TOTAL COST:**			**11,544.21**

**Table 7:** Shelter framing cost using coco lumber.

Parts	Material	Quantity	Unit Price (Php)	Price (Php)
Stilt	2 x 4 × 6′ coco lumber	9	165	1485
Side post	2 x 4 × 8′ coco lumber	6	165	990
Mid post	2 x 4 × 10′ coco lumber	3	180	540
Girder	2 x 5 × 8′ coco lumber	6	210	1260
Joist	2 x 4 × 8′ coco lumber	24	165	3960
Girt	2 x 4 × 8′ coco lumber	6	165	990
Brace	2 x 3 × 10′ coco lumber	6	115	690
Bdwalk	2 x 4 × 8′ coco lumber	10	165	1650
Bdwalk	2 x 5 × 10′ coco lumber	2	220	440
Fastener	4″ common wire nails	1	128.89	128.89
Fastener	3″ common wire nails	3	131.7	395.1
Fastener	2″ common wire nails	2	137.89	275.78
Pvc conn.	1/8″ pvc connector gusset plates (fabricated)	6	753.22	4519.32
Guywire	3/16″ nylon wire	100	1.28	128
Duct tape	Duct tape	2	265.55	531.1
**TOTAL COST:**				**17,983.19**

**Table 8:** Shelter framing cost using galvanized iron pipes.

Material	Quantity	Unit Price (Php)	Price (Php)
Galvanized Iron (GI) Pipe 1/2″ Ø (for posts, beams,	28.8	1033.85	4962.48
Galvanized Iron (GI) Pipe 3/4″ Ø (connectors)	1.0	225.4	225.4
**TOTAL COST:**			**5187.88**

**Table 9:** Shelter framing cost using bamboo.

Material	Quantity	Unit Price (Php)	Price (Php)
Bamboo, minimum of 100 mm Ø (for posts, beams)	85	20.85	1772.11
Bamboo, minimum of 75 mm Ø (arch roofing)	10	20.85	208.48
Bamboo, minimum of 50 mm Ø (arch roofing)	25	20.85	521.21
Common Wire Nails(5″)	1	235	235
Common Wire Nails (0.5″ to 1.0″)	1	151.68	151.68
**TOTAL COST:**			**2888.45**

As observed, there is high variance in the total cost of each shelter, ranging from as low as Php 2888.49 (bamboo) to Php 54,778.53 (CFS). The cost (Php/m^2^/month) of each shelter was then assessed, which is dependent on the shelter area and shelter lifespan. Table 10 displays the summarized values of cost, floor area, lifespan, and cost in Php/m^2^/month.

**Table 10:** Cost sustainability assessment and comparison.

No.	Shelter Design	Cost (Php)	Floor Area (m^2^)	Lifespan (months)	Cost (Php/m^2^/month)
1	Cold-Formed Steel	54,778.53	9.00	360	16.91
2	PVC Shelter	11,544.21	9.00	60	21.38
3	Coco Lumber	17,983.19	23.04	36	21.68
4	GI Pipe	5187.88	9.00	60	9.61
5	Bamboo	2888.48	9.00	12	26.75

The bamboo shelter has the lowest framing cost among all of the shelters but has the highest cost (Php/m^2^/month) due to its lower lifespan of 1 year. On the other hand, the GI Pipe design has the lowest cost (Php/m^2^/month) value, entailing good cost-sustainability compared to the other shelter design, which can be attributed to the low cost of the shelter materials.

The cold-formed steel shelter design comes in second with a value of 16.91 Php/m^2^/month, with the PVC shelter and coco lumber designs next in ranking. The CFS shelter, although exhibiting high price in material costs, has a long lifespan of 360 months or 30 years [52], whereas the other shelter designs only last for 12–60 months, or 1–5 years. Hence, its higher cost in comparison to a PVC-based shelter can be supported by its longer length of use. The coco lumber shelter design, while having a significantly larger floor area, has a shorter lifespan of 36 months.

### 5.3 Phase 3 – shelter implementation plan

#### 5.3.1 Planning

The planning stage takes place before the occurrence of a catastrophe. In this stage, various activities, including all coordination with the stakeholders, are done in preparation for the deployment and usage of the emergency shelter. For example, local government units can initiate the identification of areas where the shelter can be built should the need arise. This will significantly lessen the amount of time required until the shelter can be fully built. Prior to the deployment of the shelter, it is suggested to group all the members and components to easily identify which specific part will be used first once the actual assembly begins.

The first step is to find a specific location where the shelter can be properly anchored. The emergency shelter is specifically designed to be built on flat surfaces with firm and preferably on new or existing concrete pad foundations. In this regard, it is important to avoid areas where the soil is already highly saturated and muddy. Additionally, if possible, the location is preferably clear of any broken or unfinished structures that may pose a hazard.

Once the location has been identified, the front or entrance of the shelter must be positioned opposite the direction where the wind blows. This will lessen the overall wind forces acting on the structure as it hits the sloping part of the roof.

#### 5.3.2 Deployment and assembly

**Fig. 14:**
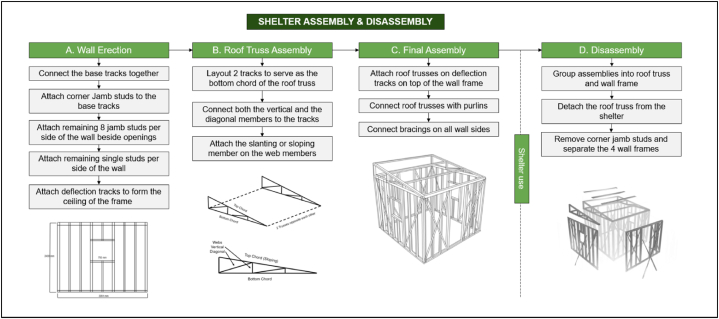
Assembly and disassembly instructions of the temporary shelter.

The deployment and assembly phase begins immediately when people are displaced by calamities, as this will serve as their primary temporary shelter. Transportation occurs from the storage facility to the shelter erection site. The logistics will depend on the number of displaced people, the location of the stored shelters, and the setup site. The factors to be determined include: (1) the number of shelters needed; (2) the amount of manpower required; (3) the transportation method; (4) the space required for the shelters; and (5) the cost. Once the logistics aspect has been determined, the shelter assembly commences. Fig. 14 shows the instructions for the assembly and disassembly of the temporary shelter.

To ensure the proper assembly of the temporary shelter, detailed instructions are presented in Table 11.

**Table 11:**
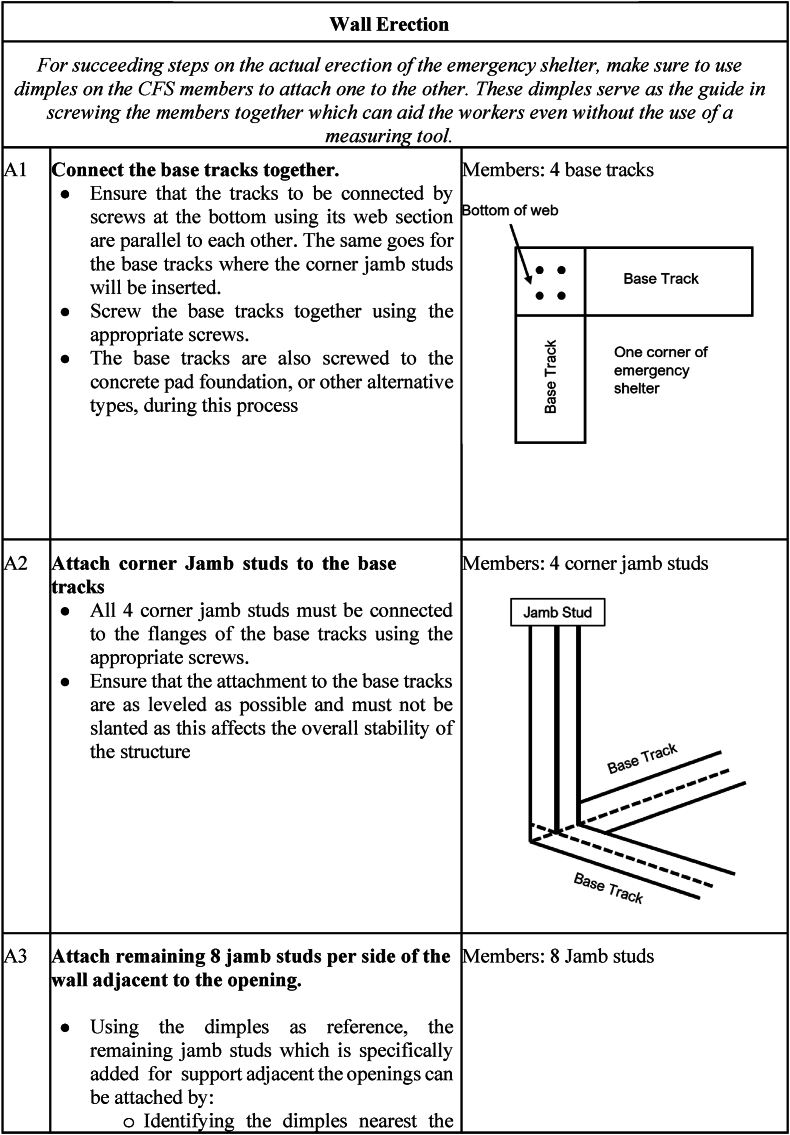

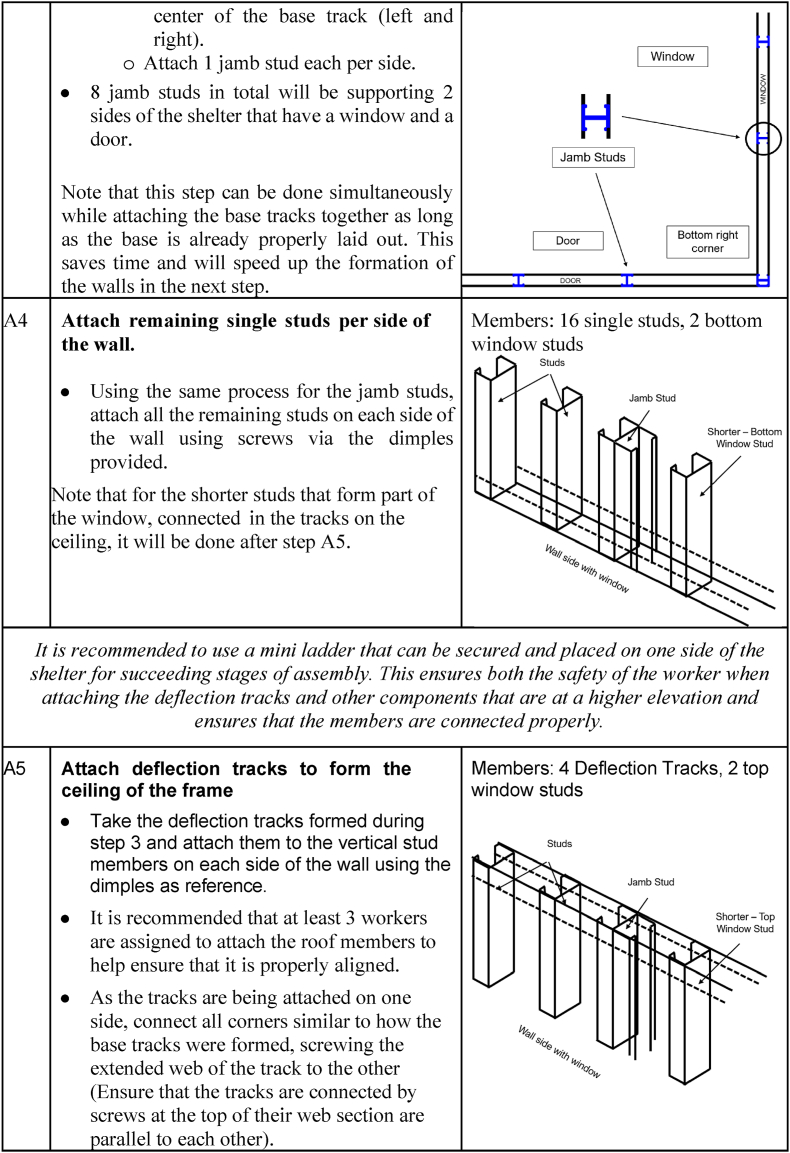

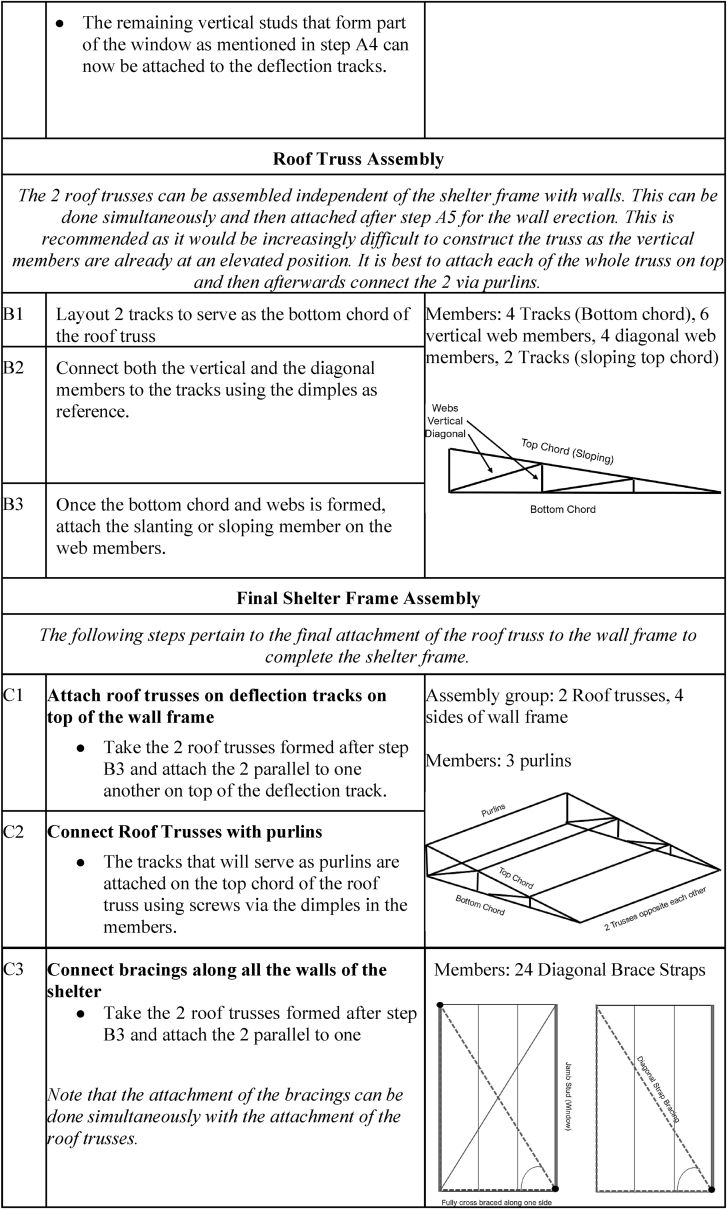
Detailed shelter assembly instructions.

Once the final shelter frame assembly is accomplished, it is best to do inspections and a round check of all the attached members and components to ensure that everything is properly attached and there are no loose ends or screws that may pose a threat to those who will be using the shelter. Once the shelter is checked, workers can proceed to place the coverings on each corresponding side of the frame.

#### 5.3.3 Disassembly

**Fig. 15:**
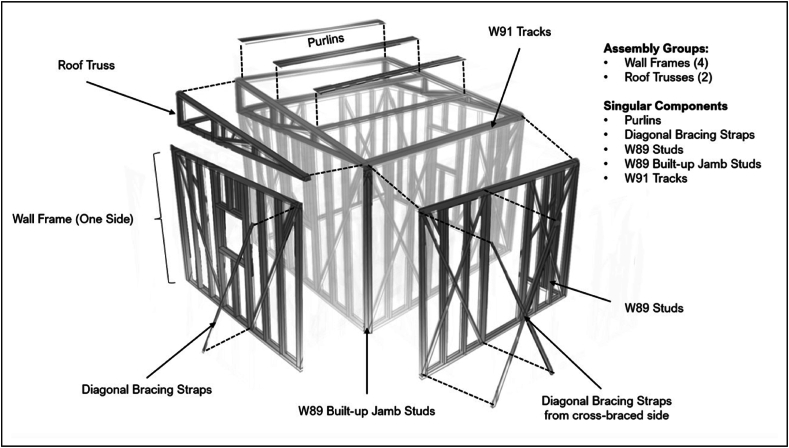
Exploded shelter view of the components and groups for disassembly.

Disassembly is a necessary phase after the utilization period of the shelters in preparation for the storage phase. The degree of disassembly will determine the net quantity of components and consequently the storage space required. Fig. 15 illustrates the exploded shelter view of the components and groups for disassembly.

The first step of disassembling the temporary shelter is to group the components into roof truss and wall frame in the same manner as erecting the shelter. Once the components are grouped accordingly, the roof truss may now be detached from the shelter. The purlins must first be detached from the wall frame through unscrewing the 3 purlins connecting the 2 roof trusses. Then, the bottom chord of the roof truss, or the tracks connected to the deflection tracks of the wall frame may now be unscrewed. After doing this, the two trusses are already detached from the wall frames and can be disassembled separately by starting from the top chord, followed by the web.

Once the roof truss has been successfully dismantled, the wall framing on the four sides may be separated. Since the two base tracks in the corners are connected by two screws at their webs, these can be removed first to detach one wall side from the other. After doing so, all four sides of the wall can be disassembled simultaneously, starting with the bracings, followed by the deflection tracks, studs, and jamb studs.

All studs, tracks, tarp covers, and screws must be recovered when disassembling for future usage. Materials should also be inspected for damage in order to provide replacement.

#### 5.3.4 Storage

The storage phase comprises of transportation to the storage facility, storage of the CFS shelter components in the facility, and inspection for safety.

Storage of cold-formed steel is necessary outside of shelter usage to avoid contact with adverse conditions. The recommended storage size may vary depending on the disassembly. Fully disassembled shelters may require less area to store, but more time to assemble during emergencies, and vice versa. Additionally, other factors to consider when identifying the specifications of a storage facility are the location of storage, available space, floor type, quantity of storage items, and frequency of movement.

Possible storage systems for the disassembled CFS shelters include rack systems and free-standing systems inside the covered structures. These storage methods are applicable to the types of materials to be stored, particularly long and narrow members, like jamb studs and deflection track, that are vulnerable to toppling when stacked together. Applicable forms of storage under racks are toast racking, cantilever racking, ladder racking, stillages, cradles, etc. For the members that are long and broad, such as base tracks and column studs, applicable forms of storage are toast racking, cantilever racking, and free-standing.

When storing the materials, it is important to keep the material away from strong acids, calcium hypochlorite, halogens, and strong oxidizers, as these chemicals may corrode the metal or initiate chemical reactions that produce harmful substances that may trigger spontaneous reactions.

Lastly, routine safety inspections are vital in ensuring that the storage environment is safe for steel. Preventing storage damage to the components is one of the factors to ensure the long-term lifespan of the shelter as well as cost-effectivity.

## 6 Conclusion and recommendations

### 6.1 Conclusion

The research focused on developing and optimizing cold-formed steel framing for emergency shelters based on multiple references for the use of the CFS material with wind loads as the governing load analyzed for member deflections. The structure is limited specifically to the optimization of the shelter frame which includes a wall frame and roof truss. Design criteria and parameters are referenced from standardized codes and previous studies concerning the structural analyses of shelters used in disaster response. Structural modeling makes use of Midas gen software with a multi-stage iterative approach to limit the excessive member movement in the frame. The process for identifying required connections is adopted from existing studies and codes to account for the necessary amount and type to be used. The final frame design is then costed using commercially available prices provided by Accutech Philippines and assessed for sustainability via the cost in Php/m^2^/month.

The study aimed to address gaps in the Philippines' existing disaster shelter plans, particularly the need for designs that can withstand post-typhoon wind loads without requiring placement in roofed locations. The final shelter design, developed through iterative modeling, utilized five components optimized to withstand design wind loads and provide post-typhoon shelter. With the lack of a standard for currently accepted and used emergency shelters in the country, the research aims to contribute to the body of knowledge through the following conclusions.

• Iterative Modeling Stages and Member Deflections
  Adding 2 studs along each side of the wall decreases the overall lateral deflections of the shelter by 0.01 m in both directions (x and y).
  Adding member bracings, specifically placed diagonally, and increasing the uniform member thickness of all members significantly reduced the maximum deflection values.
  Maximum deflection results and deflection values of nodes in the shelter can be further decreased by adding diagonal members.
  The governing load combinations that caused the maximum deflections on members of the shelter across all stages and configurations included wind forces acting completely perpendicular toward one side of the shelter frame.
  Higher deflection values closer to 1/400 of the building height induced lesser stresses on the members.
• Connection Design
  A total of 260 screw connections were found to be sufficient based on a conservative design.
  The best size for the screws was obtained based on the tension pull-out limit state for the corner connections. The same screw size was used for all the connections of the shelter to simplify the actual construction process.
  The CFS shelter was structurally designed to resist wind speeds of up to 156 mph while sustaining deflection allowed by the NSCP. Additionally, its ease of assembly and disassembly are also considered for the design, allowing for simple and faster mobilization and transportation, outdoor application, and reusability in other locations.
• Cost Sustainability
  The most to least cost-sustainable shelter designs in Php/m^2^/month is as follows: (1) GI Pipe (2) CFS; (3) PVC shelter; (4) Coco Lumber; and (5) Bamboo.
  The proposed CFS shelter, despite having the highest cost, still ranks second in terms of cost sustainability among the other less costly traditional shelters.
  On a wider perspective, the use of CFS as a primary framing material can provide ease in material fabrication and structure installation, better strength-to-weight ratio, versatility in application, and reusability.
  While GI pipes were found to be more cost-sustainable in terms of Php/m^2^/month that CFS shelters, the installation of GI pipes require welding which does not allow disassembly to smaller components. Additionally, GI pipes require more tools and equipment for installation, which essentially corresponds to higher installation costs than CFS shelters.
  Given that the Philippines is regularly affected by typhoons, there is a need to address the long-term sustainability of emergency shelters. As such, investment in a CFS-framed emergency shelter serves as a more cost-efficient alternative because of its capacity to provide utility for up to 30 years and several other significant advantages in terms of structural strength and reusability, in contrast to the traditional alternatives that have a lower cost but limited lifespan and performance.

### 6.2 Recommendations

To further enhance this study, the following are recommended.

• Engage stakeholders, such as local government units (LGUs), communities, and non-government organizations (NGOs), through conducting stakeholder perception surveys to improve the post-disaster shelter design and integration into existing frameworks.
• Use the proposed design as a basis for upgrading to a permanent structure and conduct in-depth studies on foundation, roofing, and wall components.
• Explore worst-case loading scenarios, including perpendicular wind directions and seismic loads, to optimize the shelter for stronger forces and multiple disasters.
• Compare shelters with identical features, such as wind resistance and ease of mobilization, for a more accurate cost-sustainability analysis. Consider structural integrity, environmental impact, and other sustainability factors beyond cost alone.
• Expand structural design and analysis of cold-formed steel shelters to extend their reach to countries susceptible to tropical cyclones.

The proposed shelter falls under the classification of an emergency shelter, which is reflected in the structural design and its properties. In this study, the design process was limited mostly to the body of knowledge available from existing literature studies, codes, and design references. A persisting challenge is to be able to design a shelter that is not only structurally viable but also centers on humanitarian challenges for post-disaster situations. To further integrate methods to address this, it is recommended that for future research, coordination with stakeholders, most importantly the local government units, affected communities, and humanitarian relief non-government organizations (NGOs), will be done to be able to properly address persisting problems for post-disaster shelters conditions and to be able to either properly integrate or seek for improvements on the currently implemented framework for post-disaster shelters. More than this, the introduction of CFS as a framing component poses promising results, especially with the advantages it can provide. As there are existing studies that tackle the use of CFS as framing material for transitional shelters, where permanency for a longer period is an option, the researcher suggests that future researchers use the proposed design In this study as a basis for research on improving the structural design of the shelter where there is an option for upgrade to a permanent structure using the same material components, or with minimal improvements. More than this, given that the study focuses on the framing design, the researchers also recommend that future researchers incorporate a more in-depth study on other shelter components, specifically on the foundation design, roofing cover, and wall members or shelter covering.

## CRediT authorship contribution statement

**Daniel Nichol Valerio:** Writing – review & editing, Writing – original draft, Validation, Supervision, Software, Resources, Project administration, Methodology, Investigation, Funding acquisition, Data curation, Conceptualization. **Cheryl Lyne Roxas:** Writing – review & editing, Supervision, Project administration, Investigation, Data curation, Conceptualization. **Kenneth Jae Elevado:** Writing – review & editing, Visualization, Validation, Project administration, Investigation, Formal analysis, Data curation. **Jeremy Brian Branzuela:** Writing – original draft, Methodology, Investigation, Formal analysis, Data curation. **Desiree Dale Chua:** Writing – original draft, Visualization, Methodology, Formal analysis, Data curation. **Gabriel Lambatin:** Writing – original draft, Visualization, Methodology, Formal analysis, Data curation. **Gian Carlo Tiu:** Writing – original draft, Visualization, Methodology, Formal analysis, Data curation.

## Data and code availability

Data will be made available on request.

## Declaration of competing interest

The authors declare that they have no known competing financial interests or personal relationships that could have appeared to influence the work reported in this paper.
